# Metagenomic Sequencing for Surveillance of Food- and Waterborne Viral Diseases

**DOI:** 10.3389/fmicb.2017.00230

**Published:** 2017-02-15

**Authors:** David F. Nieuwenhuijse, Marion P. G. Koopmans

**Affiliations:** Department of Viroscience, Erasmus Medical CenterRotterdam, Netherlands

**Keywords:** viral pathogens, metagenomics, high-throughput sequencing, surveillance, foodborne

## Abstract

A plethora of viruses can be transmitted by the food- and waterborne route. However, their recognition is challenging because of the variety of viruses, heterogeneity of symptoms, the lack of awareness of clinicians, and limited surveillance efforts. Classical food- and waterborne viral disease outbreaks are mainly caused by caliciviruses, but the source of the virus is often not known and the foodborne mode of transmission is difficult to discriminate from human-to-human transmission. Atypical food- and waterborne viral disease can be caused by viruses such as hepatitis A and hepatitis E. In addition, a source of novel emerging viruses with a potential to spread via the food- and waterborne route is the repeated interaction of humans with wildlife. Wildlife-to-human adaptation may give rise to self- limiting outbreaks in some cases, but when fully adjusted to the human host can be devastating. Metagenomic sequencing has been investigated as a promising solution for surveillance purposes as it detects all viruses in a single protocol, delivers additional genomic information for outbreak tracing, and detects novel unknown viruses. Nevertheless, several issues must be addressed to apply metagenomic sequencing in surveillance. First, sample preparation is difficult since the genomic material of viruses is generally overshadowed by host- and bacterial genomes. Second, several data analysis issues hamper the efficient, robust, and automated processing of metagenomic data. Third, interpretation of metagenomic data is hard, because of the lack of general knowledge of the virome in the food chain and the environment. Further developments in virus-specific nucleic acid extraction methods, bioinformatic data processing applications, and unifying data visualization tools are needed to gain insightful surveillance knowledge from suspect food samples.

## Introduction

Transmission via the food- and waterborne route is a common mode of spread of a wide range of viruses. Many commonly recognized food- and waterborne infections are caused by viruses that are transmitted by the fecal-oral route. Particularly caliciviruses (norovirus, sapovirus) can cause diarrhea and vomiting and less commonly astroviruses, rotaviruses, and adenoviruses (Newell et al., 2010). Other viruses cause symptoms resulting from extra-intestinal spread, like hepatitis A (HAV), and hepatitis E (HEV). High levels of viral shedding through stool and vomit lead to dispersal in the environment. Moreover, the stability of many food- and waterborne viruses allows for prolonged persistence in the environment. Food- and water associated transmission is also suspected to enhance the spread and emergence of zoonotic viruses (e.g., Middle East Respiratory Syndrome-coronavirus and Nipah virus) and facilitates the occurrence of zoonotic events though the handling of bushmeat (Ebola virus) (Wolfe et al., 2005; European Food Safety Authority, 2014; Mann et al., 2015).

Challenges of detecting viruses transmitted by the food- and waterborne route are their diversity and the frequent secondary person-to-person transmissions, which may mask an initial food- or waterborne introduction. In addition, there is a lack of awareness among clinicians (Beersma et al., 2012), as the symptoms caused by foodborne viruses are not specific to the viruses causing the illness. Furthermore, there is limited coverage in surveillance of food- and waterborne viral disease, hampering detecting and tracing (Ahmed et al., 2014; Verhoef et al., 2015).

In the past years, high-throughput sequencing technologies have increased the ability to measure genomic material from diverse samples tremendously. These methods will most likely continue to improve in the future (Aarestrup et al., 2012). Specifically, metagenomic analysis using untargeted sequencing has received a lot of attention, because the high throughput of current sequencing technologies has made it possible to obtain multiple high coverage genomes from highly complex samples (Cotten et al., 2014; Smits et al., 2015). Even though it is still a developing field, metagenomics is starting to become mature enough for applications outside of the research environment.

With the development of multiplex real-time polymerase chain reaction (RT-PCR) protocols came the realization that unraveling etiologies of main disease syndromes is more complex than previously recognized. This led to questions about the detection of viruses for which the role as causes of illness remains to be evaluated, the importance of co-infections and recognition of less common disease etiologies (Binnicker, 2015). Similarly, high throughput metagenomic sequencing broadens the scope of detectable viruses, which, apart from making it more complex, make us further understand the role of viruses in health and disease. The biggest promise, however, is that of routine application of metagenomic sequencing in diagnostic context, facilitating viral detection and offering huge potential for tracing of viruses in (foodborne) outbreaks.

## Recognizing Food- and Waterborne Viral Disease

**FIGURE 1:**
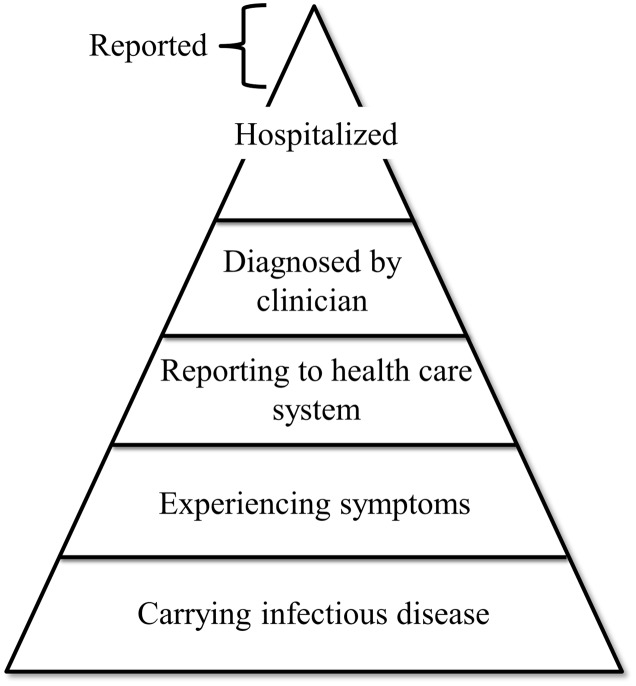
**Schematic representation of the phenomenon known as the “surveillance pyramid”.** Layers represent different categories of infected individuals. Width of the layers represents the estimated number of individuals in that category. As indicated, individuals reported by surveillance programs generally originate from the hospitalized category.

Given the number of different viral pathogens potentially associated with food- and waterborne transmission their detection has not been straightforward. Partly because many of these pathogens lack cell culture systems that are sensitive and robust enough for application in routine settings (Amar et al., 2007). The entry point for disease-based surveillance of viruses spreading by food and water is the reporting of patients presenting to a clinician. However, patients only present themselves in case of a severe symptomatic infection, or in case self-help is not sufficient. Mild symptoms are therefore generally not registered creating a bias in surveillance. This phenomenon is captured in the surveillance pyramid (**Figure 1**), and the full extent of disease can only be captured through epidemiological studies addressing incidence and etiology at community level coupled with severity of a range of enteric pathogens (Sethi et al., 1999; de Wit et al., 2001; Tam et al., 2012). Additionally, it is challenging to distinguish between foodborne outbreaks and outbreaks caused by direct contact between humans. Classic clinical symptoms of foodborne disease vary, ranging from diarrhea and vomiting to abdominal cramps and general malaise, which makes it hard for clinicians to pinpoint the exact causative agent. This leads to misdiagnosis if the diagnostic workup is selective, and if there are no obvious signs of food-related exposure (Beersma et al., 2012). Moreover, heterogeneity in clinical interpretation can be caused by host factors, such as differences in the expression of histo-blood-group antigens that are receptors for rota- and noroviruses (Payne et al., 2015; de Graaf et al., 2016). Susceptibility to fecal-orally transmitted viruses may also be influenced by the established microbiome and virome in the host population, of which the prior is shown to differ between different locations and age groups (Yatsunenko et al., 2012). It is reasonable to think that the differences in the gut environment are more pronounced between countries with larger social and economic differences such as first and third world countries, which often differ in their resident pathogens (Ott et al., 2012; Yatsunenko et al., 2012; Hay et al., 2013). The role of the gut virome, in addition to the gut microbiome, is a relatively new concept and has been described as potentially having influence on gut health and therefore expression of disease (Cadwell, 2015). Because of under and miss-diagnoses, clinical surveillance likely only captures the tip of the iceberg of food- and waterborne viral disease cases.

## Detection of Food- and Waterborne Viral Disease Outbreaks

In cases where a cluster of patients with similar symptoms presents itself, there can be an investigation to look for epidemiological clues of the link between the cases. Additional information is garnered from the use of viral genome sequencing, making it possible to track origins of outbreaks, and to estimate how much of the observed human disease is attributable to foodborne infection by computerized linking of epidemiologic data to aligned viral genomic sequences (Verhoef et al., 2011). However, often the original source or evidence of it being food- or waterborne cannot be found, which means that outbreaks often are merely registered. Of the 941 viral disease outbreaks reported as foodborne in the joint ECDC-EFSA surveillance report of 2015, only 9.1% had robust evidence of food- or waterborne transmission (Eurosurveillance editorial team, 2015). Routine application of genotyping of HAV in newly diagnosed cases quadrupled the number of cases in which food was the most likely source of infection a 3 year enhanced surveillance study in The Netherlands, but this is not commonly done (Petrignani et al., 2014). In an investigation of 1794 food- and waterborne outbreaks in Korea, roughly 75% of the outbreaks reported in schools and public restaurants were attributed to an unknown origin (Moon et al., 2014). Availability and costs of molecular testing combined with sequencing, additional to the limited success of virus detection in food products, are likely further limiting their use in food and water surveillance. This is demonstrated by the fact that formal confirmation of a viral outbreak associated with food- and waterborne transmission still requires extensive epidemiological analysis or confirmation of a virus in the infected individual, or both (ESFA, 2016). However, due to the increase of genomic information of viruses, sequence data is increasingly used to support and strengthen outbreak investigations. Nevertheless, the surveillance programs for these viruses in the human food chain is limited, in contrast with the American CDC^1^ and the European ECDC^2^ surveillance programs for bacteria and parasitic pathogens causing food- and waterborne diseases (Deng et al., 2016) and does not have widespread coverage. As an example, to comply with European food safety regulations, shellfish, a well-known source of foodborne pathogens, need to be tested for enteric bacteria. However, it has been well documented that shellfish that pass quality control based on bacterial counts may still contain human pathogenic viruses (Rodriguez-Manzano et al., 2014). To be able to recognize food- waterborne viral disease outbreaks and stop underestimation of its disease burden there should be innovations in the current foodborne surveillance system.

## Classical Viruses Associated with Food- and Waterborne Diseases

Although the list of viruses causing acute gastroenteritis is long, norovirus ranks among the top causes of diarrheal disease (Ahmed et al., 2014). Reporting of outbreaks suggests that the food- and waterborne disease transmission route is relatively rare, but provides an underestimate, bearing in mind that it may be hard to recognize a food- and waterborne transmission route in community-acquired diarrheal disease. To quantify the burden of all diarrheal disease attributable to foodborne transmission, the World Health Organization commissioned a study that combined data from surveillance and exhaustive literature reviews with a systematic approach to calculation of the fraction of disease attributable to food contamination (Havelaar et al., 2015). This ranked the burden of norovirus illness among the top causes of foodborne disease, along with *Campylobacter*, and listed HAV associated disease among other significant causes of foodborne disease, along with *Salmonella* and *Taenia solium*.

For bacterial foodborne pathogens, the analysis of systematically collected surveillance data has been used as the basis of attribution analysis (Pires et al., 2009). A popular approach has been to quantify the proportion of foodborne disease of humans to their likely origin, by comparing diversity of strains found in human disease outbreaks with that found in animal and environmental reservoirs (Hald et al., 2007). While this model does not allow estimating the foodborne disease where food is a vehicle for person-to-person transmission, which is common for noroviruses, it has been used with some success to quantify the contribution of foodborne viral disease stemming from environmentally contaminated food (e.g., associated with shellfish; (Verhoef et al., 2015)). This builds from the observation that there is a large discrepancy between the norovirus variants in clinical settings and environmental samples (Tao et al., 2015; Kazama et al., 2016). Norovirus GII.4, found in clinical setting, is generally related to person-to-person transmission, however, several other norovirus genotypes and genogroups were found in environmental samples in the same area. However, food associated acute gastroenteritis is not limited to norovirus infections. In a large retrospective study of oyster-related acute gastroenteritis outbreaks in Osaka City in Japan 30.7% of the cases were attributed to other pathogens such aichivirus, astrovirus, sapovirus rotavirus A, and enteroviruses (Iritani et al., 2014). Furthermore, outbreaks can be caused by a mixture of these viruses and viral variants (Wang et al., 2015).

## Other Viruses Transmitted via the Food- and Waterborne Route

Apart from viruses causing gastroenteritis, there are viruses causing food- and waterborne diseases that are associated with a variety of other syndromes. The second most common disease syndrome is hepatitis, caused by HAV, a fecal-orally transmitted virus (Havelaar et al., 2015). By decreasing natural exposure in regions with low endemicity, the susceptibility of the population for outbreaks of HAV disease in these regions is increasing (Newell et al., 2010). Because of increased globalization, contamination of food products by viruses prevalent in food producing regions can increase the risk of outbreaks in these regions. Several outbreaks of HAV infection have been reported in recent years both in the USA and Europe (Gossner et al., 2014). Most of these outbreaks could be identified as foodborne infections after intense investigations (Bruni et al., 2016). Especially fresh (imported) food products (e.g., fresh frozen berries, pomegranate seeds, and sun-dried tomatoes) have been identified as sources of the virus (Gossner et al., 2014; Tavoschi et al., 2015). Tracking the foodborne source of infection is challenging for HAV, because of an underestimation of the contribution of food as a source of infection due to the long incubation period in infected individuals (Petrignani et al., 2014).

Another foodborne virus gaining increased attention is zoonotic HEV, associated with genotype 3 and 4 HEV. HEV is widespread in commercially held pigs, as well as in wild pigs, and deer (Guillois et al., 2016). Human disease with genotype 3 HEV is increasingly recognized, but in the large majority of the cases the source of the virus is unknown (Lewis et al., 2010). There is clear evidence that food can be a source of zoonotic HEV infections. Outbreaks that have been confirmed to be caused by foodborne transmission of the virus by consumption of wild meat from boar, deer, and rabbit (Tei et al., 2003; Izopet et al., 2012; Guillois et al., 2016). Several studies have shown the zoonotic potential of HEV from pigs (Teixeira et al., 2016), HEV can also be readily detected in pork products such as dried meats and liver sausages (Di Bartolo et al., 2015). A large proportion of food-related HEV infections, however, does not lead to hospitalization of the patient, leading to under-reporting and unrecognized risk and burden of the disease (Guillois et al., 2016).

Beside viruses circulating in livestock, wildlife has the potential to be a large reservoir of unknown zoonotic viruses. Hunting, trading, preparing, and consuming so-called “bushmeat” is one of the routes by which novel viruses can be introduced into the human population (Karesh and Noble, 2009). It may be difficult to disentangle foodborne infection from direct zoonotic exposure, but it is important to consider local practices before ruling out food as a source of human infection. A special example are the occasional introductions of Nipah viruses from bats into humans through contamination of date palm sap which is collected in open containers to which bats that harbor these viruses have access (Rahman et al., 2012). Not proven but certainly interesting is the practice of drinking unprocessed camel urine which may contain MERS coronavirus, a practice that came to light during the investigations into sources of MERS coronavirus infection in humans (Funk et al., 2016). Even if limited in scale, small foodborne infections, originating from human-wildlife interaction, constitute as many incidents potentially pushing wildlife viruses to become human-to-human transmissible (Wolfe et al., 2005; Islam et al., 2016). In the cases of Monkeypox and Nipah this only led to small epidemics, but when the virus is well adapted to spread from human to human this can lead to larger outbreaks, as seen during the Ebola crisis in 2015 (Wolfe et al., 2005; Mann et al., 2015). Continuing deforestation, increasing population and continued trade of bushmeat brings more humans in contact with wildlife and increases the risk of zoonosis (Karesh and Noble, 2009). Urbanization and globalization of travel and trade provides ample and increasing opportunity for further spread. Therefore, even anecdotal zoonotic introductions may constitute a public health risk, and ideally should be investigated in conjunction with the animals these humans were exposed to. As the ability to spread between humans is a key property for successful further spread, enhancing the capacity to investigate clusters of disease (in humans and animals) is important (McCloskey et al., 2014).

## Unknown Fecal-Oral Passengers

Bacteriophages, although not directly pathogenic to humans, could play a role in human health and disease by influencing the gut microbiome. Sequencing data from human gut samples presents a large diversity of bacteriophages in the human gut (Reyes et al., 2012). In addition to bacteriophages, untargeted sequencing of sewage samples has shown the presence of large quantities of different plant viruses (Zhang et al., 2006). Because of the presence of numerous infectious plant virus particles in human fecal waste there is ongoing research on the effect of these viruses in human health and disease (Colson et al., 2010). Similarly, there is ongoing research into the impact of bacteriophages on human health through their modulating effect on the gut microbiome (Reyes et al., 2012), and thereby, gut immunity (Honda and Littman, 2016). In what way bacteriophages protect or expose the human gut to bacterial or viral pathogens has yet to be further investigated. However, using metagenomic sequencing it will at least be possible to recognize the presence of unknown fecal-oral passengers.

## Metagenomics for Food- and Waterborne Viral Disease Surveillance

Metagenomics is a term used for experiments in which all nucleic acids in a certain sample are sequenced. For bacteria, historically, the diversity of a sample used to be expressed by performing phylogenetic analyses based on 16S ribosomal RNA (Handelsman, 2004). However, since viruses lack such a universally conserved motif, viral metagenomics refers to the attempt to recover full and partial genomes of all viruses present in the sample. Viral metagenomic analysis protocols generally start with procedures to remove host and bacterial cells followed by nuclease treatment to remove free nucleic acids. Often, the remaining nucleic acids are amplified using randomly primed (RT-) PCR and finally sequenced using high-throughput sequencing technology. Viral metagenomics has great potential in surveillance of viruses in the global food chain because of its sensitivity, broad detection range, and detailed information of the detected virus (Aarestrup et al., 2012).

### Environmental Surveillance

**FIGURE 2:**
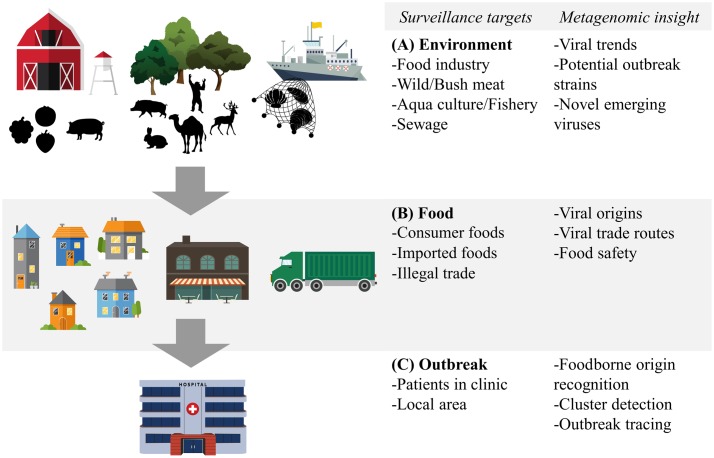
**Food- and waterborne viral surveillance targets for metagenomic sequencing approaches.** **(A)** Environmental surveillance of food industry, wild meat and bushmeat habitat, and aquaculture and fishery environment. **(B)** Food surveillance of consumer and imported foods, including illegally imported foods. **(C)** Surveillance of food- and waterborne outbreaks, in clinic and locally. Potential of metagenomic sequencing based surveillance is listed next to each category.

Metagenomic sequencing has already been used in the sampling of the world’s oceans to estimate the global viral diversity (Hingamp et al., 2013). Similarly, metagenomics can be used in environments associated with viruses spread via the food- and waterborne route (**Figure 2A**), which gives an overview of all these viruses and circumvents the mentioned sampling biases. The potential of such an approach for food-related purposes was exemplified by Hellmér et al. (2014) who conducted a multi-species viral surveillance study and, albeit not metagenomic sequencing based, were able to detect several food- and waterborne viruses in sewage. Interestingly, norovirus and HAV, detected in sewage, could be related to hospitalized patients diagnosed with the viral infection in the catchment area of the sewage system. Moreover, they detected a peak in the level of norovirus several weeks before the outbreak was reported in the hospital in that area (Hellmér et al., 2014). This demonstrates the potential power of shifting the scope of surveillance of food- and waterborne viruses from the hospital to the environment. Untargeted metagenomic sequencing has been shown to be able to capture a multitude of viruses in sewage samples in several studies. Moreover, comparison between sewage viromes from Nigeria, Nepal, Bangkok, and California, four geographically distant locations, showed distinct differences in the subsets of detected human viruses (Ng et al., 2012). Interestingly, the average sequence similarity between the reference sequences stored on the NCBI GenBank and the human viruses detected in the samples from California was higher than those from the other locations. This may indicate a bias towards American viruses in view of human virus diversity in this database (Ng et al., 2012). A study that looked at viruses from sewage capable to infect human epithelial cells was able to detect a large number of bacteriophages and several different species of the *Polyomaviridae. Picornaviridae*, and *Papillomaviridae* viral families.(Aw et al., 2014). Another more recent evaluation of untargeted metagenomic sequencing for surveillance purposes retrieved full genomes of Adeno-associated virus-2 as the most prominent mammalian virus in the sample. This virus is generally not associated with any pathology and cannot be grown in cell cultures, possibly underestimating its role in diarrheal disease (Furtak et al., 2016). A striking fact of these studies is the number of sequencing reads that are found that share no sequence similarity with current reference databases. Percentages of unmapped sequences range from 37 to 66% (Cantalupo et al., 2011; Ng et al., 2012). Whether these sequences represent novel viruses that can be transmitted via the food- and waterborne route remains to be determined. Nevertheless, these preliminary studies show the potential of untargeted metagenomic sequencing to detect novel and known human pathogens. Sampling a larger variety of locations, performing longitudinal studies of the same environment and deeper sequencing will provide more information on what environmental metagenomic sequencing can contribute to the monitoring of viral trends and viral diversity.

### Food Surveillance

Analogous to the environment in which it has been produced, food itself can benefit from metagenomic surveillance. Food contamination in combination with international trade, changing eating habits and food processing practices all contribute to the spread of food- and waterborne viruses and making food itself a valuable target of metagenomic surveillance (**Figure 2B**). Sentinel screening of imported foods, especially risk foods such as fresh fruits and vegetables, dried meats and seafood, could prevent foodborne viral outbreaks such as the international HAV outbreak in Europe from 2012 to 2013 (Severi et al., 2015). Successful application of metagenomic sequencing of viruses has been shown in a study isolating viruses in the family of *Reoviridae* and *Picobirnaviridae* from field-grown lettuce (Aw et al., 2014).

Apart from legal trade, illegal import of food products, such as bushmeat, could also be screened. Untargeted metagenomic sequencing is especially suited for these types of screenings, as the origin and the potential viral content of these samples are often completely unknown. In one example, metagenomic sequencing was performed on bushmeat seized by the customs officers of a French airport. Although no viruses with a potential threat to human health could be detected (Temmam et al., 2016), these initial attempts should be looked at as potentially interesting surveillance approaches, given that relatively large quantities of raw bushmeat are estimated to enter Europe and the Americas annually (Mann et al., 2015).

Another source of known and potentially unknown foodborne disease-causing viruses are shellfish. Mainly the consumption of oysters is associated with foodborne outbreaks (Bellou et al., 2013). However, oysters, cockles, and clams have been shown to accumulate norovirus, sapovirus, and HAV (Benabbes et al., 2013). To our knowledge, there are no published studies performing untargeted virome sequencing of these shellfish. Surveillance by metagenomic sequencing can be beneficial for aquaculture, also for monitoring seafood health, as in aquaculture, large numbers of animals are kept in a confined environment for an extended period, increasing opportunities for the spread of infections. Cultivated fish and other sources of seafood can be infected with a wide variety of viruses (Alavandi and Poornima, 2012).

### Outbreak Surveillance

One of the main promises of surveillance using metagenomic sequencing is that of concomitant clinical application (diagnosis of patients) and public health application (typing and cluster analysis to trace of food- and waterborne outbreaks) (**Figure 2C**). Using metagenomic sequencing, the effort of detecting and genotyping of a virus can be combined to trace an outbreak, regardless of prior knowledge of the virus, provided the data is analyzed in combination with relevant metadata. The use of this integrative approach has been demonstrated in an investigation of a hospital outbreak of human parainfluenza virus, which was investigated using high-throughput metagenomic sequencing (Greninger et al., 2016). Both the detection of the virus, the diagnosis of the disease and the establishment of viral clusters and transmission routes could be derived from the metagenomic sequencing data. A similar approach should enable investigation of viruses related to food- or waterborne diseases and distinguishing between a food- and waterborne and a person-to-person transmission route. In such investigations, speed is of the utmost importance, therefore on-site sequencing strategies, enabled by novel portable sequencing platforms such as the Oxford Nanopore MinION (Hoenen et al., 2016), have potential in fast local outbreak detection and disease monitoring (Arias et al., 2016). Recent reports have shown potential in metagenomic detection of hepatitis C, chikungunya, Ebola and Zika virus in hospital settings (Greninger et al., 2015; Sardi et al., 2016). The development of on-site sequencing technology is still in its infancy, however, and it remains to be investigated if food-related viral outbreaks will be traceable and can deliver whole-genome based viral dynamics analysis analogous to the investigation of the Ebola outbreak of 2014 (Gire et al., 2014; Quick et al., 2016). However, the same on-site technology has been shown to be beneficial in tracing foodborne salmonella (Quick et al., 2015). Aspects of current on-site sequencing technologies that need to be improved for viral metagenomic sequencing are the limited throughput and sequence quality, which limit the detection of low-level viral genomes and minor variants. Nevertheless, the use of near-real-time sequencing of Ebola and Zika during the recent outbreaks has received a lot of attention and has shown that the technology works.

## Challenges in Sample Preparation and Sequencing

The routine application of metagenomic sequencing for clinical diagnosis and surveillance is dependent costs versus performance criteria such as speed, reliability, and comparability of results with those of reference methods. Improvements are necessary in the standardization and speedup of sample preparation, sequencing and data analysis for clinical and public health application. A recent study has shown the potential of fast whole-genome sequence based epidemiological tracing in the recent Ebola outbreak (Arias et al., 2016). However, specific primers were used to target the Ebola genome, which is different from a metagenomic sequencing approach.

The developments and different choices of sequencing technology make it difficult to decide how to standardize routine diagnostics and surveillance protocols. Studies that directly compare platforms help in this decision making process. Two studies compared the Illumina MiSeq, Roche-454 titanium, Ion Torrent PGM, and PacBio RS platforms (Quail et al., 2012; Frey et al., 2014). For viral metagenomics application, the Illumina and the Ion Torrent platform seem to outperform the other two platforms based on their relatively low cost per giga base output. Between these two systems, the main tradeoff is the sequencing time versus the sequencing read output. The high volume of sequencing reads produced by the Illumina platform, in a longer timeframe, increases the chance that a lowly abundant viral genome is sufficiently covered, which makes it more suitable for metagenomics of complex samples. The Ion Torrent platform delivers a smaller number of reads in a smaller timeframe, which is beneficial when a timely result is necessary, and low level presence of viruses is disregarded, for instance in diagnostic settings.

Novel approaches, such as the MinION nanopore sequencer, increase speed and depth of coverage at the cost of sequence error rate. A comparison of a metagenomics approach using the MinION nanopore or the Illumina MiSeq sequencer reports a sample-to-result time of 6 h for a MinION nanopore setup compared to 20 h using an Illumina MiSeq setup (Greninger et al., 2015). Despite their reported successes in identification of viruses, the reported error rate of 10 to 60 percent impedes high resolution sequence classification at low genome coverage, or the use of sequence data for reliable source-tracking. It does allow very rapid virus classification in cases where low coverage suffices, or at high viral titers (Hoenen et al., 2016; Quick et al., 2016). However, performance of the MinION sequencing platform remains to be tested at lower virus titers and with more complex samples which are generally encountered in surveillance and clinical settings (food, feces, sewage).

To increase the viral specific output of metagenomics sequencing approaches sample preparation methods can be used to reduce non-viral genomic material or specifically select for viruses. Approaches that are being investigated, range from different extraction protocols (Cotten et al., 2014; Conceição-Neto et al., 2015) to using a virome specific capturing chip (Briese et al., 2015) or blood-derived antibodies to capture viral particles (Oude Munnink et al., 2013). Paradoxically, however, the sequencing capacity of high throughput metagenomic sequencing is sensitive enough to pick up contaminants from the lab reagents, or from previous experiments (Gruber, 2015). These pose a challenge to the interpretation of metagenomic data. To limit contamination, laboratories in which samples are processed are often separated from those in which nucleic acids are amplified and equipment is UV treated and cleaned with bleach. Additionally, alternating the sample-specific DNA barcodes in multiplex sequencing experiments reduces contamination from previous runs. Nevertheless, it is recommended to include both negative control samples, which have been processed similarly, but are believed to contain no viruses, and positive control samples, that contain known quantities of a variety of viruses (Lusk, 2014). Alternatively, bioinformatics tools such as DeconSeq (Schmieder and Edwards, 2011), have been developed to check for signals of regularly found lab contamination in the sequencing data. In conclusion, as contamination of samples and equipment may not be avoidable, its likelihood should be taken in consideration when using metagenomic sequencing technology for food-related surveillance applications.

## Difficulties of Metagenomic Data Analysis

Aside from lab-based technical difficulties, there are several challenges concerning data analysis of metagenomic sequencing experiments. First, due to the high and increasing read output of sequencing machines, data analysis of high throughput sequencing projects generally requires strong computational infrastructure, which, can require large investments and technological expertise (Spjuth et al., 2016). However, metagenomic data analysis tools have been improving, optimizing the ratio between computing resources needed and their speed and accuracy. Sequence annotation tools based on k-mer lookup tables such as UBLAST (Edgar, 2010), Kraken (Wood et al., 2014), Kaiju (Menzel et al., 2016), and Diamond (Buchfink et al., 2014) have increased the speed of sequence assignment to reference database with several orders of magnitude, while requiring relatively modest processing power.

Second, the assembly of millions of genomic fragments into 1000s of different individual genomes is a daunting task. Historically, short-read assemblers were developed and optimized to assemble a single genome out of a set of sequencing reads. These assemblers are therefore not suited for the reconstruction of metagenomes and are prone to creating synthetic chimeric genomes (Vázquez-Castellanos et al., 2014). Various assemblers have since been developed specifically aimed at metagenome assembly, like MetaSPAdes (Nurk et al., 2016), Ray-Meta (Boisvert et al., 2012), MetAMOS (Treangen et al., 2013), MetaVelvet (Afiahayati et al., 2015), and IDBA-UD (Peng et al., 2012). Nevertheless, metagenome assembly is still a challenging task, often requiring manual editing to resolve miss-assemblies.

Third, assigning all assembled genomes to a reference genome is hampered by miss-annotations and incomplete reference databases. One example is “non-A, non-B hepatitis virus”, a sequence present in the NCBI GenBank, which was miss-annotated and the sequence was shown to belong to a bacteriophage (Cantalupo et al., 2011). The volume of sequencing databases is increasing rapidly, however, sequence annotations and metadata are of varying levels of quality and the speed of analysis decreases with increasing reference datasets. Therefore, there is a tradeoff between the rate of success of annotation of a sequence against a smaller curated reference dataset, and reliability of annotation using a large reference database with less-well curated annotation data.

Sequence homology of multiple reference genomes can lead to the spurious assignment of sequencing reads to one of these genomes. An example of the impact of spurious read annotation was the alleged detection of genomic material of *Yersinia pestis* in the New York subway system. Further inspection showed that the reads mapping to *Yersinia pestis* could have mapped with similar likelihood to other bacterial species (Afshinnekoo et al., 2015). Such miss-annotations of metagenomic sequences need to be anticipated and carefully addressed before using metagenomics in surveillance and diagnostic applications.

## Metagenomic Data Interpretation

The final challenge of metagenomic sequencing based surveillance is the interpretation of the annotated sequences. There is still little knowledge of the presence and dynamics of viruses in the environment and the food chain, which is of influence on the interpretation of food- and waterborne viral surveillance samples. Various factors are expected to influence the virome, and without knowledge of the typical viral content of a sample, the relevance of the detection of a virus is hard to determine. An example of this is a study showing a large discrepancy between the levels of HAV genotypes detected in sewage samples compared to the genotype infecting patients in the clinic in the same time (La Rosa et al., 2014). A potential sampling bias and asymptomatic shedding of one of the variants was proposed as an explanation of the discrepancy. However, this shows that a lack of knowledge of viral diversity in a population under surveillance could potentially lead to wrong conclusions in environmental surveillance studies.

Detection of a virus by molecular methods relies on intact genomic material of a virus being present in the sample. However, the relationship between the infectivity of a detected virus and the detection of a fragment of its genome is not unambiguous. Apart from intrinsic virus characteristics, infectivity and detection of a virus depends on its stability in the sample matrix (Cook and Rze, 2004) and during sample preprocessing steps (Conceição-Neto et al., 2015). Similarly, the detection of a virus using untargeted metagenomic sequencing does not confirm its infectivity. Cell culture based infectivity essays are the golden standard to determine virus infectivity, these methods are, however, not scalable and many viruses cannot be cultured *in vitro* (Hamza et al., 2011). High genome coverage combined with close sequence identity to a viral reference genome with a known pathogenic phenotype are currently the strongest links between metagenomic sequencing data and disease etiology. Nevertheless, currently employed PCR based methods, which are based on genome fragment detection, suffer from the same limitations (D’Agostino et al., 2011).

It is becoming increasingly clear that integration of different data sources and experimental results is crucial for the interpretation of metagenomic sequencing experiments. Therefore, browsing of these data and visualization of relationships between genome datasets and metadata should be facilitated. In the recent years, interactive web-based data browsing and visualization tools have increased in popularity to facilitate the interaction with and the browsing through highly complicated data in a user-friendly manner. Further development of tools that facilitate interaction with and visualization of metagenomic sequencing results, such as Kronatools (Ondov et al., 2011) and Taxonomer (Flygare et al., 2016), and frameworks for so-called data analysis “dashboards”^3,^^4,^^5^, should make the interpretation of metagenomics experiments easier in the future.

## Conclusion

In our current society, there is much attention for the diseases that are causing occasional outbreaks. However, there are multiple strong signs that there are viruses hiding below the radar, due to a focus on viruses with direct clinical impact. As such, the disease burden of food- and waterborne viral infections is mainly recorded in outbreaks, signified by severe symptoms and hospitalization. However, it is estimated that the large abundance of viral infections causing mild symptoms, and thus not being recorded, carry a large portion of the global food- and waterborne disease burden. Moreover, this disease burden is expanded by the consequential infections and outbreaks of these viruses in susceptible populations. Global food trading, diversification of food sources and interactions with animals and other reservoirs of food- and waterborne disease related viruses complicate the capability of investigators to detect the original source and to determine the transmission pattern of viruses causing foodborne outbreaks. Therefore, surveillance efforts should look to metagenomic sequencing technologies, bioinformatics analysis tools and data sharing initiatives to get a more realistic insight in the global burden of food- and waterborne viral disease, and to make informed decisions on how to reduce this burden.

## Author Contributions

DN and MK designed the focus of the review, DN did the literature search and drafted the manuscript, MK reviewed and revised.

## Conflict of Interest Statement

The authors declare that the research was conducted in the absence of any commercial or financial relationships that could be construed as a potential conflict of interest.

## Footnotes

1 http://ecdc.europa.eu/en/healthtopics/food_and_waterborne_disease/surveillance/Pages/index.aspx

2 http://www.cdc.gov/ncezid/dfwed/keyprograms/surveillance.html

3 http://shiny.rstudio.com/

4 https://plot.ly/

5 http://jupyter.org/
